# Two-pore channel regulators - Who is in control?

**DOI:** 10.3389/fphys.2024.1534071

**Published:** 2025-01-10

**Authors:** Rebecca Deutsch, Veronika Kudrina, Marc Freichel, Christian Grimm

**Affiliations:** ^1^ Walther Straub Institute of Pharmacology and Toxicology, Faculty of Medicine, Ludwig-Maximilians-University, Munich, Germany; ^2^ Institute of Pharmacology, Heidelberg University, Heidelberg, Germany; ^3^ DZHK (German Centre for Cardiovascular Research), Partner site Heidelberg/Mannheim, Heidelberg, Germany; ^4^ Immunology, Infection and Pandemic Research IIP, Fraunhofer Institute for Translational Medicine and Pharmacology ITMP, Munich/Frankfurt, Germany

**Keywords:** lysosome, TPCS, TPCN1, TPCN2, JPT2, LSM12, Rab7a, TMEM63a/b

## Abstract

Two-pore channels (TPCs) are adenine nucleotide and phosphoinositide regulated cation channels. NAADP activates and ATP blocks TPCs, while the endolysosomal phosphoinositide PI(3,5)P_2_ activates TPCs. TPCs are ubiquitously expressed including expression in the innate as well as the adaptive immune system. In the immune system TPCs are found, e.g. in macrophages, mast cells and T cells. In cytotoxic T cells, NAADP activates TPCs on cytolytic granules to stimulate exocytosis and killing. TPC inhibition or knockdown increases the number of regulator T cells in a transmembrane TNF/TNFR2 dependent manner, contributing to anti-inflammatory effects in a murine colitis model. TPC1 regulates exocytosis in mast cells *in vivo* and *ex vivo*, and TPC1 deficiency in mast cells augments systemic anaphylaxis in mice. In bone marrow derived macrophages NAADP regulates TPCs to control phagocytosis in a calcineurin/dynamin dependent manner, which was recently challenged by data, claiming no effect of TPCs on phagocytosis in macrophages but instead a role in phagosome resolution, a process thought to be mediated by vesiculation and tubulation. In this review we will discuss evidence and recent findings on the different roles of TPCs in immune cell function as well as evidence for adenine nucleotides being involved in these processes. Since the adenine nucleotide effects (NAADP, ATP) are mediated by auxiliary proteins, respectively, another major focus will be on the complex network of TPC regulatory proteins that have been discovered recently.

## 1 Introduction

Two-pore channels (TPCs) are regulated by adenine nucleotides. NAADP (Nicotinic acid adenine dinucleotide phosphate) acts as an agonist and ATP (Adenosine triphosphate) as an antagonist of the two mammalian TPCs TPC1 and TPC2. Remarkably, both NAADP and ATP act on TPCs in an indirect manner, i.e. their effects are mediated through auxiliary proteins. By contrast, activation of TPCs by PI(3,5)P_2_ (Phosphatidylinositol 3,5-bisphosphate) is through direct binding on the channel. Both, NAADP and PI(3,5)P_2_ can also act together in a synergistic manner, as demonstrated for TPC2 recently (Yuan et al., 2022). When applied simultaneously, NAADP and PI(3,5)P_2_ (or synthetic analogues mimicking their effects) strongly promote Ca^2+^ release through TPC2. As mentioned, the NAADP effect on TPCs requires the presence of NAADP binding proteins. Candidates are the cytosolic proteins JPT2 (also called HNL1), Lsm12 and recently a third candidate was suggested: aspartate dehydrogenase domain-containing protein (ASPDH) (Gunaratne et al., 2021; He et al., 2022; Roggenkamp et al., 2021). In addition, other direct interaction partners and functionally interfering proteins have been discovered recently to up- or downregulate the activity of TPCs. In this review, we will discuss 1. The latest on TPC associated proteins, regulating their function and activity (Chapter I and II) and 2. The function of TPCs, their regulators and adenine nucleotides in the context of immunity and inflammation (Chapter III).

## 2 Chapter I: proteins mediating effects of adenine nucleotides on TPCs

In this chapter the following adenine nucleotide (AN) and auxiliary protein pairs regulating TPC activity will be discussed: mTOR/ATP, JPT2/NAADP and Lsm12/NAADP.

It has long been unclear how NAADP activates TPCs, and whether TPCs are activated by NAADP at all had been a matter of debate for years (Brailoiu et al., 2010; Calcraft et al., 2009; Cang et al., 2013; Gerndt et al., 2020a; Gerndt et al., 2020b; Gunaratne et al., 2018; Lin-Moshier et al., 2014; Roggenkamp et al., 2021; Ruas et al., 2015; Sakurai et al., 2015; Schieder et al., 2010; Wang et al., 2012; Yuan et al., 2022; Zhang et al., 2021; Zong et al., 2009). Since 2020/2021 however, with the discovery of lipophilic small molecules (TPC2-A1-N and TPC2-A1-P) mimicking effects of NAADP and PI(3,5)P_2_, respectively and the discovery of several NAADP/TPC binding proteins, the hypothesis of TPCs being *bona fide* targets of NAADP, albeit indirectly through NAADP binding proteins has gained some considerable momentum. Thus, Roggenkamp et al. (2021) and Gunaratne et al. (2021) showed independently from each other that NAADP binds to the cytosolic protein JPT2. While Roggenkamp et al. claimed a role of this complex (NAADP/JPT2) in ryanodine receptor 1 (RyR1) activation in T cells, confirming direct interaction by co-immunoprecipitation experiments, Gunaratne et al. (2021) and Gunaratne et al. (2023) showed, also in co-immunoprecipitation experiments interaction of JPT2 with TPCs. In the same year, Zhang et al. (2021) showed Lsm12 to interact with NAADP and with both TPC1 and TPC2 (for more detailed reviews see Guse, 2022; Krogsaeter et al., 2021; Marchant et al., 2022; Patel et al., 2021; Walseth and Guse, 2021). Gunaratne et al. (2023) confirmed furthermore selective binding of both JPT2 and Lsm12 to NAADP via radioligand binding assays and biolayer interferometry, and the interaction between JPT2 and Lsm12 with TPCs. In a pseudoviral trafficking assay, Lsm12 KO and JPT2 KO showed the same phenotype as TPC1 KO and TPC2 KO. This was the first evidence that both JPT2 and Lsm12 are involved in endolysosomal trafficking events similar to TPC1 and TPC2. In 2022 a third protein, aspartate dehydrogenase domain-containing protein (ASPDH) was reported as an NAADP-binding protein (He et al., 2022), awaiting however further functional confirmation.

In sum, an important step towards a better understanding of how NAADP interacts with TPCs seems to have been made.

**FIGURE 1:**
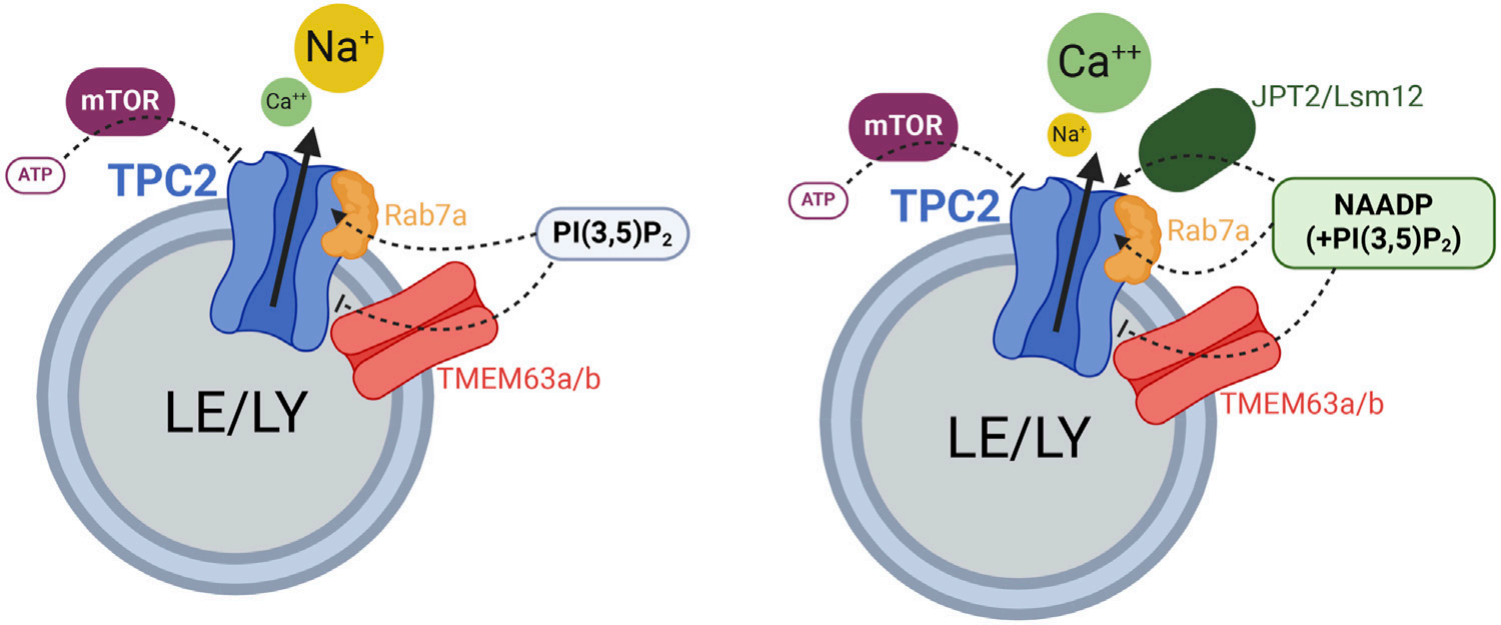
Cartoon displaying the different regulators of TPC2 function. Rab7a enhances TPC2 activity while TMEM63a/b (OCaR1/2) reduce TPC2 activity. ATP blocks TPC2 through mTOR. JPT2 or Lsm12 mediate NAADP activation of TPC2. Created with BioRender.com.

Much less controversial than the NAADP effect is the effect of ATP on TPCs. ATP through binding to mTOR (mammalian target of rapamycin), which itself binds to TPCs (confirmed by co-immunoprecipitation experiments) strongly inhibits TPC activity (Cang et al., 2013; Chao et al., 2017). Thus, two AN, NAADP and ATP differentially regulate TPC activity, binding themselves to auxiliary proteins that mediate their effects on TPCs by direct interaction (Figure 1).

Importantly, it should be stressed that on the one hand, TPCs act as non-selective Ca^2+^-permeable channels when activated by NAADP. On the other hand, TPCs are clearly highly-selective Na^+^ channels when activated by the endolysosomal phosphoinositides, PI(3,5)P_2_, PI(3)P (TPC1) and/or by voltage (TPC1). The recently discovered small molecule activators TPC2-A1-P and TPC2-A1-N support these findings as the former renders the channel more selective for Na^+^ while retaining some Ca^2+^ permeability, whereas the latter significantly increases the channel’s Ca^2+^ permeability.”

## 3 Chapter II: other protein regulators of TPC activity

In this chapter we will discuss additional recently discovered and functionally validated interactors of TPCs: Rab7a, which binds TPC2 and enhances its activity (Abrahamian et al., 2024) and TMEM63A, which functionally antagonizes the activity of TPC1 and TPC2 channels and NAADP mediated Ca^2+^ release from TPC2 channel containing organelles, therefore also termed organellar Ca^2+^ regulator (OCaR1) (Freichel et al., 2024; Tsvilovskyy et al., 2024) (Figure 1).

More than 70 Rab proteins are known in mammalian genomes. Rabs play fundamental roles in intracellular trafficking and vesicle formation, vesicle movement, and fusion. Rabs are localized to the cytoplasmic face of intracellular vesicles and organelles. While Rab4 and 11 are involved in fast and slow recycling in the recycling endosomal (RE) pathway, Rab5 is predominantly found in early endosomes (EE), and Rab7 localizes to late endosomes (LE) and lysosomes (LY). Two Rab7 isoforms exist, Rab7a and Rab7b, sharing about 50% sequence similarity. Rab7a controls vesicular transport to LE and LY in the endocytic pathway and plays fundamental roles in the biogenesis, positioning, and motility of lysosomes, auto- and phagolysosomes. Rab7a also plays roles in autophagy (regulating fusion with lysosomes), phagocytosis, cell survival, cell migration, growth, differentiation and apoptosis. In contrast, Rab7b localizes to LE as well as the trans-Golgi network (TGN) and controls vesicular transport from endosomes to the TGN (Bucci et al., 2010; Vestre et al., 2021). Rab7b promotes the degradation of toll-like receptor 4 (TLR4) and can negatively regulate the inflammatory activation of macrophages. However, in contrast to Rab7a Rab7b is not involved in the regulation of the EGF/EGFR degradation pathway, shown before to depend on TPC2 activity (Grimm et al., 2014; Müller et al., 2021). Of note, in a recent report Rab7b was claimed to interact with TRPML1, another endolysosomal cation channel (Vestre et al., 2021). However, functional data for this interaction are missing, i.e. patch-clamp electrophysiology or GCaMP based Ca^2+^ imaging data to corroborate any functional interaction between Rab7b and TRPML1. Rab7a instead was tested in endolysosomal patch-clamp experiments and found not to have an effect on TRPML1 activity (Abrahamian et al., 2024). Modulation of Rab7a activity affects a number of disease pathologies including Niemann Pick type C1 (NPC1), Charcot-Marie-Tooth type 2B, infectious diseases and cancer. Lin-Moshier et al. had already shown in 2014, as part of a proteome/interactome analysis that Rab7a interacts with TPC2, confirmed by co-immunoprecipitation experiments. Abrahamian et al. (2024) have now corroborated this finding by an extended FRET analysis and co-immunoprecipitation experiments, but importantly also provided endolysosomal patch-clamp evidence to support a functional effect of Rab7a on TPC2 channel activity. Thus, Rab7a strongly increased TPC2 activity, independent of the agonist applied, i.e. PI(3,5)P_2_, or the above mentioned lipophilic small molecule agonists TPC2-A1-N (mimicking the effect of NAADP) or TPC2-A1-P (mimicking the effect of PI(3,5)P_2_). Further proof of an acute effect of Rab7a on TPC2 was provided by the application of the Rab7 inhibitor CID1067700 in the endolysosomal patch-clamp configuration, which instantly abrogated the effect. Importantly, in endogenously expressing cells knockout (KO) of Rab7a resulted in reduced TPC2 currents. Nevertheless, more work is needed to assess whether Rab7a indeed directly interacts with TPC2 or whether it interacts via additional proteins in a larger complex. Likewise, it remains to be elucidated whether other Rab proteins regulate the function of TPCs or interact with TPCs.

As a pathophysiological consequence of the Rab7a mediated effect on TPC2 activity, proliferation, migration and invasion of melanoma cells were found to be reduced after Rab7 KO or knockdown (KD), similar to TPC2 KO or KD. In addition, Rab7 KO phenotypes could be rescued with TPC2 but not *vice versa*, suggesting that Rab7a is an upstream regulator of TPC2, with the latter one being the target protein eventually affecting proliferation, migration and invasion (Abrahamian et al., 2024).

In analogy to these findings, it is assumed that Rab7a mediated upregulation of TPC2 activity (TPC1, was not found to interact with Rab7a) would likewise be relevant in immune cells as both proteins are widely expressed in the immune system. Interestingly, LRRK2 (Leucine-rich repeat kinase 2), which was previously shown to co-immunoprecipitate with TPC2 (Gómez-Suaga and Hilfiker, 2012) reportedly also interacts with Rab7a (Liu et al., 2022); see also review by Gómez-Suaga and Hilfiker (2012). What effect LRRK2 has on the activity of TPC2 remains unclear, likewise it remains unclear how TPC2 activity is affected if both LRRK2 and Rab7a are present.

In contrast to Rab7a, Tsvilovskyy et al. (2024) have recently shown that mammalian OCaR1/TMEM63A antagonizes the activity of both TPC channels. TMEM63 proteins (TMEM63A, TMEM63B, TMEM63C) are the closest mammalian homologues of the family of *Arabidopsis* OSCA proteins. OSCA1.1 was identified in 2014 in an *Arabidopsis* screen, in which a mutant (*osca1*) exhibited a reduced hyperosmolarity-induced [Ca^2+^]_i_ increase (Yuan et al., 2014). Yuan et al. showed that OSCA1.1 represents a plasma membrane protein forming hyperosmolality-gated calcium-permeable channels. In the same year, membrane proteins encoded by the *Arabidopsis* gene *At4G22120* (AtCSC1 or OSCA1.2) were identified as cation channels activated by hyperosmotic shock, and the human orthologue HsTMEM63C (*HsCSC1*) could also form an osmotically-gated calcium conductance (Hou et al., 2014). The *Arabidopsis* OSCA family has 15 members, and phylogenetic analysis across the taxa revealed 11 OsOSCA members in rice (*Oryza sativa)* with OsOSCA1.4 localized in the plasma membrane and nine other OsOSCA proteins in the endoplasmic reticulum (ER) (Zhai et al., 2020). In *Drosophila*, one orthologue was described (DmTMEM63), which was reported to be important for the activation of neurons in the tongue by mechanical stimuli and the ability to choose food based on particle size (Li and Montell, 2021) and humidity sensing (hygrosensation) in olfactory neurons, a process that is evoked by changes in osmolality (Li et al., 2022).

Osmotic forces are one of the triggers for activation of mechanosensitive channels, and in 2018 Murthy et al. revealed that several members of the OSCA family represent bona-fide mechanosensitive ion channels as they could demonstrate that expression of AtOSCA1.1 and AtOSCA1.2 in PIEZO1-deficient HEK cells gave rise to slowly or non-inactivating high-threshold non-selective cationic mechanosensitive currents in the plasma membrane evoked by both cell-membrane indentation as well as following pressure application to a recording pipette (Murthy et al., 2018). Reconstitution experiments in liposomes showed that AtOSCA1.2 is gated directly by changes in membrane tension and thus intrinsically mechanosensitive. In parallel, Zhang et al. showed that AtOSCA1.1 and AtOSCA3.1 1 are mechanosensitive channels, and they found that they form symmetric homodimers (Zhang et al., 2018), which was also reported for OsOSCA1.2 (Maity et al., 2019), whereas mammalian TMEM63A, TMEM63B and TMEM63C were reported to form monomeric channels (Qin et al., 2023; Zhang et al., 2023; Zheng et al., 2023). Interestingly, expression of some AtOSCA proteins (AtOSCA1.8, AtOSCA2.3, AtOSCA3.1) as well as *Drosophila* (DmTMEM63) and mammalian orthologues (MmTMEM63A, MmTMEM63B, MmTMEM63C, HsTMEM63A) mediated currents evoked by pressure application via the patch pipette, but not by cell indentation (Murthy et al., 2018). Yet, measured currents were surprisingly small, i.e. in the low pA range (Murthy et al., 2018). While in plants OSCA proteins were shown to be hyper-osmolarity activated, in mammals TMEM63 proteins are reportedly sensitive to both hyper- and hypoosmolarity (Du et al., 2020; Yang et al., 2024). Du et al. (2020) further reported that TMEM63B is localized in outer hair cells (OHCs) of the inner ear. OHCs lacking TMEM63B fail to maintain cell shape under hypotonic stress and loss of TMEM63b leads to hearing loss in mice.

A lethal respiratory failure was observed when both TMEM63A and TMEM63B were deleted specifically in AT2 cells due to impairment of lung-inflation-induced Ca^2+^ oscillations and subsequent surfactant release causing failure in alveolar expansion (Chen et al., 2024). Stretch-activated currents, which could not be activated by cell membrane indentation, carried mainly by K^+^ and Na^+^ but not Ca^2+^ or Mg^2+^, were negligible in the plasma membrane of AT1/AT2 cells lacking both TMEM63A and TMEM63B, and totally abolished in that of laminar bodies (LBs) of these cells, a specialized lysosome-related organelle storing ATP and surfactant. Since TMEM63B was mainly localized at the limiting membrane of LBs in AT2 – but could not be visualized at the plasma membrane under basal conditions according to analysis of a mouse line with a knock-in of a VA-tagged TMEM63B protein, it can be concluded that fusion of LBs with the plasma membrane can account for the presence of small amounts of TMEM63B and pressure-dependent currents at the cell surface (Chen et al., 2024).

Tsvilovskyy et al. (2024) also found a predominant localization of TMEM63A/OCaR1 in the membrane of intracellular organelles using a high-resolution organellar proteomics approach. In pancreatic acinar cells, TMEM63A/OCaR1 was localized between intracellular (mainly endo-lysosomal/secretory) vesicles and the plasma membrane, indicating its dynamic subcellular (re-) distribution during endo-/exocytosis. Using a genetically encoded Ca^2+^ indicator targeted directly to Two-pore channel 2 (TPC2)-containing vesicles, it could be shown that TMEM63A/OCaR1 controls Ca^2+^ release from NAADP-responsive, acidic Ca^2+^ stores under basal conditions and upon stimulation of GPCR. The increased Ca^2+^ release in TMEM63A/OCaR1-deficient acinar cells is the consequence of the lack of functional inhibition of endo-lysosomal TPC1 and TPC2 channels leads to exacerbation of the disease phenotype in murine models of severe and chronic pancreatitis. Because of their regulatory role in Ca^2+^ release from intracellular organelles, TMEM63A proteins were termed OCaR1 as mentioned above. Of note, in contrast to TPCs the endolysosomal Ca^2+^ release channel TRPML1 seems to be unaffected by the presence of TMEM63 proteins (Tsvilovskyy et al., 2024).

The studies described above established the concept that these proteins form cation channels in the membrane of intracellular organelles, e.g., acidic endo-lysosomes and secretory granules with a small portion of TMEM63a/OCaR1 proteins being translocated to the plasma membrane during exocytosis. Here, TMEM63/OCaR proteins regulate the Ca^2+^ release either after hormonal stimulation or mechano-activation, and thereby control important body functions and the development of life-threatening diseases such respiratory failure and severe pancreatitis.

**FIGURE 2:**
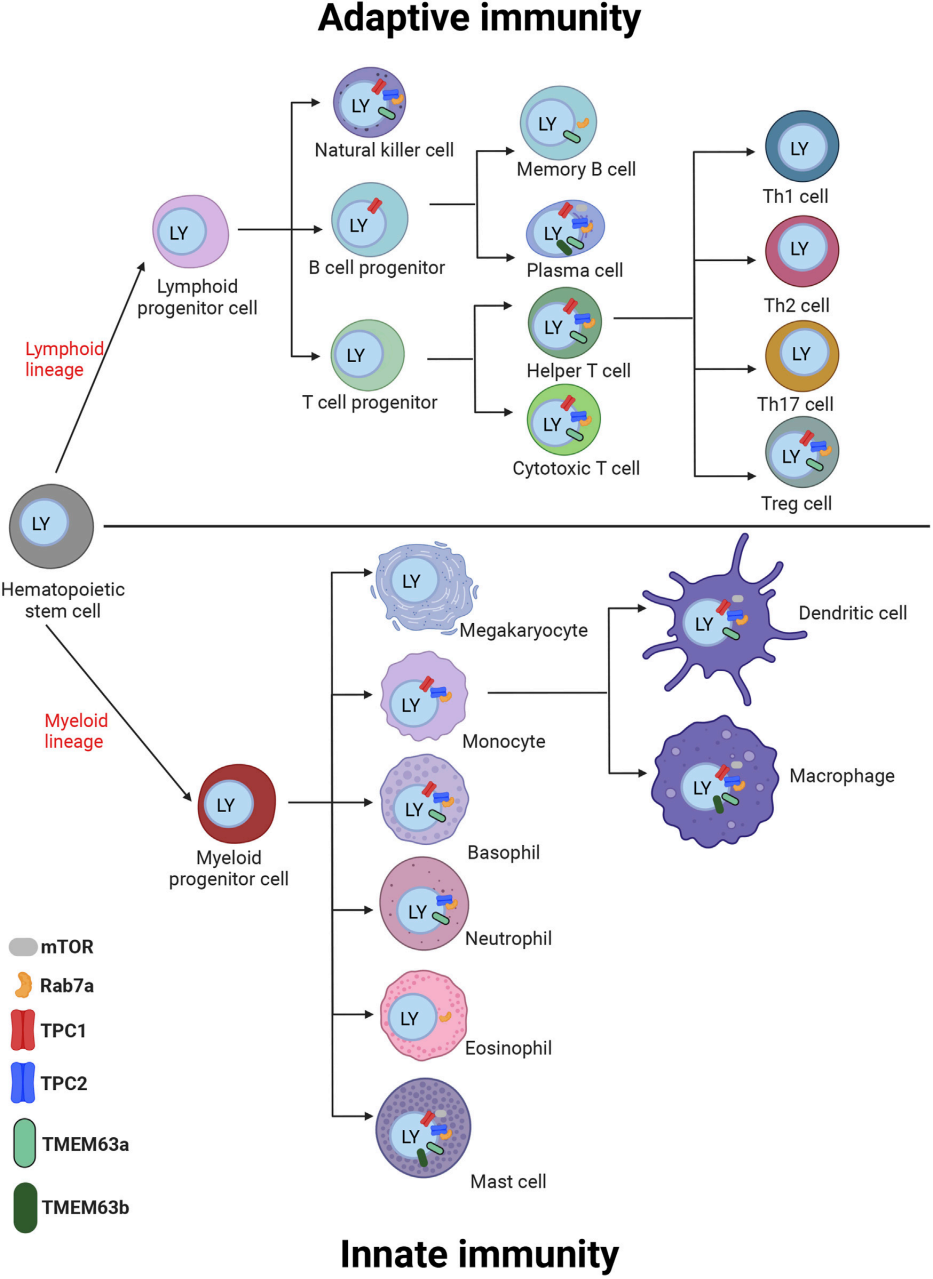
Cartoon of immune cells of the innate and adaptive immune system with expression of TPC channels, Rab7a, mTOR, and TMEM63a/b in the cells. Different experimental approaches proved the expression of the TPCs in these cells as summarized in chapter III and additionally proteins are shown on the cells with RNA expression levels above the threshold of 5 NX according to the data in Figure 3. The proteins are depicted on or in close proximity to the endosome/lysosome (blue) inside the cell. Created with BioRender.com.

**FIGURE 3:**
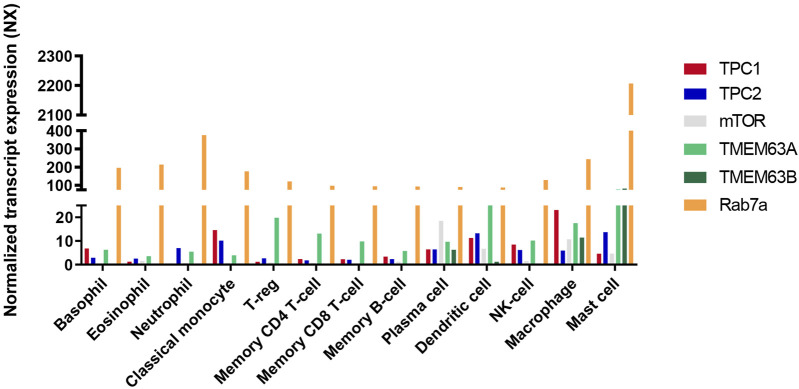
Bar diagram of gene expression levels of TPCs, Rab7a, mTOR and TMEM63a/b in human immune cells. The RNA sequencing data in various human immune cells acquired from the Human Protein Atlas (HPA) are showing the gene expression levels. The expression levels are shown as normalized expression (NX), calculated from the internal normalization pipeline for 18 different immune cell types. Based on data from Human Protein Atlas: Immune/Single Cell Expression (RNA) of TPCN1, TPCN2, mTOR, TMEM63a, TMEM63b, and Rab7a available from: https://www.proteinatlas.org).

It is expected that in cells other than pancreatic acinar cells, including immune cells TMEM63 proteins would also play similar regulatory roles controlling TPC activity, e.g., in macrophages and mast cells, which express high levels of TMEM63A and B (Figures 2, 3). Atomic absorption spectroscopy measurements in TMEM63A-deficient macrophages revealed an increase of Na^+^ and Ca^2+^ and reduction of K^+^ concentrations in lysosomes, respectively (Cai et al., 2024). This study suggests that TMEM63A is largely responsible for the mechanosensitive sodium leak from lysosomes under high hydrostatic pressure triggering micropinocytosis, which is impaired upon inactivation of TPC (but not TRPML1) channels. The impairment in organellar Na^+^ and Ca^2+^ homeostasis in TMEM63A-deficient macrophages is associated with impaired activation of TFEB/TFE3 and their nuclear translocation which depends on their dephosphorylation by the Ca^2+^-dependent phosphatase calcineurin (Cai et al., 2024).

In macrophages, it is well conceivable how the increase in hydrostatic pressure during phagocytosis and macropinocytosis activates mechanosensing TMEM63/OCaR proteins in the membrane of intracellular organelles. However, it remains a future challenge to elucidate how changes in membrane tension, acting on the cell surface from the outside, e.g. during lung inflation or after changing osmolarity, is transmitted to these organellar channels. Furthermore, it remains unknown whether and how changes in membrane tension are involved in the gating and activation of TMEM63/OCaR channels following stimulation of the cells by agonists or other chemical signals, including changes in the metabolism in acinar cells or neuro (endocrine) cells as well as many other cells in which they are expressed. An interesting finding in this direction is that AtOSCA1.1 was found to be directly activated by lyso-phosphatidylcholine (Lyso-PC), independent of mechanostimualtion (Shan et al., 2024), but this was not tested in mammalian TMEM63/OCaR proteins so far. Another recently discovered function of TMEM63 proteins is to act as a lipid scramblase localized at the PM and lysosomes (Miyata et al., 2024), activating bidirectional lipid translocation upon changes in membrane curvature and thickness. Thus, TMEM63 proteins have the potential to permeate ions and phospholipids, and it remains to be shown whether the loss of this mode of action may contribute to the regulation of TPC function.

TMEM63 loss of function mutations were described in four patients with a myelination deficit that resembles a hypomyelinating leukodystrophy, presenting with congenital nystagmus, motor delay, and deficient myelination in infants, very similar to the infantile stage of the prototype of hypomyelinating leukodystrophies, Pelizaeus-Merzbacher disease (PMD). In the affected individuals, which carry heterozygous missense variants in the pore-forming domain of TMEM63A, the myelin deficit had almost completely resolved after 4 years of age (Yan et al., 2019).

## 4 Chapter III: adenine nucleotide regulated TPCs in immunity and inflammation

In mammalian cells TPCs are found to be expressed in a range of immune cells (Figures 2, 3), e.g. in macrophages, mast cells, and in T cells (Davis et al., 2020). recently showed that in murine bone marrow-derived macrophages (BMDM) loss of TPCs results in reduced Fc-gamma receptor (FcγR) mediated phagocytosis. They further showed that this effect was NAADP dependent as well as dependent on calcineurin and dynamin. Vice versa, heterologous expression of TPC1 and TPC2 restored phagocytosis. By contrast, only the phagocytosis of large beads or particles (3.9–6 μm) but not smaller particles was affected by TRPML1, in line with previously reported results (Samie et al., 2013). Thus, according to Davis and colleagues TPCs are critical for phagocytosis over a broad range of particle sizes in macrophages. Their data imply an important role for TPCs in immune surveillance and in the innate immune response against pathogens. This finding has been challenged recently by (Chadwick et al., 2024), who claimed that TPCs are required neither for phagocytosis nor for phagosome maturation but rather regulate endomembrane tension to enable remodeling and resolution of phagolysosomes. In a further recent work, mentioned previously (Cai et al., 2024), the same group proposed that TMEM63A in lysosomes, under increased hydrostatic pressure is gated to cause lysosomal Na^+^ release, thus potentially playing an important role e.g., in lysosomal tubulation. A role of TRPML2, another reportedly mechanosensitive cation channel in endolysosomes (Chen et al., 2020) in this process was ruled out by using the TRPML inhibitor ML-SI3. Of note, only enantiopure (−)-(R,R)-trans ML-SI3 inhibits all three TRPML channels, while racemic ML-SI3 activates TRPML2 (Leser et al., 2020). In light of the functional interaction between TPCs and TMEM63A (TPC activity is reduced in presence of TMEM63 proteins as mentioned above), it remains to be investigated whether, *vice versa* the activity of TMEM63 proteins is affected by the presence of TPCs and what implications this may have for the aforementioned processes.

In another study, the role of TPC1 on histamine release in mast cells was studied. Thus, TPC1 knockout mice showed an augmented anaphylaxis and increased histamine levels in the supernatant of cultured TPC1^−/−^ peritoneal mast cells (PMCs) (Arlt et al., 2020). At the same time, the histamine content of the PMCs was found to be increased. The proposed interpretation of this data was that loss of TPC1 enhances degranulation and release of histamine due to increased Ca^2+^ content in the endoplasmic reticulum (ER). By contrast, in the presence of TPC1, the ER Ca^2+^ content and the release of histamine are regulated in a controlled manner through ER-endolysosome contact and TPC1 mediated endolysosomal Ca^2+^ release. This is a surprising finding as TPC activation is otherwise reported to enhance the release and exocytosis of endolysosomal and granular content (Davis et al., 2012; He et al., 2022; Martucci et al., 2023; Scotto Rosato et al., 2022). In addition, TPC1 is predominantly found in early endosomes and not in late endosomes or lysosomes (Castonguay et al., 2017). One might therefore also postulate that increased histamine levels in the supernatant of TPC1^−/−^ PMCs are the result of reduced reuptake of histamine (Cabut and Haegermark, 1966; Slamet Soetanto et al., 2019), with a potential role of TPC1 in this process. Future work in this direction would be needed to rule out such a possibility. Of note, TPC1, in contrast to TPC2 can also be activated by PI(3)P, a phosphoinositide mainly localized to early endosomes. In addition, in contrast to TPC2, which is largely pH independent, the activity of TPC1 is increased at pH 6.8 as found in early endosomes compared to pH 4.6 as found in late endosomes/lysosomes. In sum, this strongly argues for a relevant role of TPC1 in early endosomal function.

Besides macrophages and mast cells, TPCs are also described to be functionally active in different types of T cells. Thus, NAADP activates TPCs on T cell cytolytic granules to stimulate exocytosis and killing as reported by Davis et al. (2012). Application of NAADP-AM was further shown to evoke granzyme B secretion in a Ned-19 and bafilomycin A1 sensitive manner, pointing to acidic Ca^2+^ store involvement (Davis et al., 2012). The authors concluded that the cytolytic granules store and release the Ca^2+^ for their own exocytosis. Similarly, Martucci et al. (2023) claimed that TPCs control oxytocin secretion from the neurohypophysis. Mice lacking TPCs showed impaired maternal and social behavior due to impaired secretion and reduced plasma levels of oxytocin. However, in their model Martucci et al. (2023) propose that TPCs may provide the local Ca^2+^ signal needed for priming, a process occurring before the exocytosis of, e.g., oxytocin containing vesicles, which first need to be recruited closer to the plasma membrane in a Ca^2+^ dependent manner.

Another important question is whether modulation of TPCs affects the secretion or expression of inflammatory mediators. TPC inhibition or knockdown in mice was shown to increase the number of regulator T (Treg) cells in a transmembrane TNF/TNFR2 dependent manner, contributing to anti-inflammatory effects in a murine colitis model (He et al., 2020). Tumor necrosis factor (TNF) is initially expressed as tmTNF (= transmembrane or membrane-bound TNF) on the surface of dendritic cells, which can be cleaved by TNFγ-converting enzyme (TACE) into sTNF (= soluble TNF). TPC inhibitors prevented the release of sTNF, resulting in the upregulation of tmTNF expression, which ultimately leads to activation and expansion of highly immunosuppressive Tregs. Whether these effects are NAADP or PI(3,5)P_2_ dependent however remains currently unclear.

## 5 Conclusions and outlook

In sum, recent evidence suggests that TPCs are involved in a multitude of immune cell functions, reportedly regulating inflammatory mediator release, phagosome resolution, phagocytosis, granzyme B secretion, or histamine release. In most cases a NAADP dependence of the observed effects was proposed. NAADP, and likewise ATP effects on TPCs are mediated by auxiliary proteins: mTOR, JPT2 and Lsm12. Other functional interactors of TPCs, TMEM63 proteins and Rab7a have recently been described as positive and negative regulators of TPC activity with potential roles also in immune cells (TMEM63 proteins and Rab7a are ubiquitously expressed). Of note, most prevalent expression of TMEM63A in immune cells was demonstrated for macrophages and mast cells.

It remains unclear, at the moment whether KO of JPT2 and/or Lsm12, which are expected to abrogate the claimed NAADP dependent effects mediated by TPCs in immune and other cells, would present with similar phenotypes as described for TPC KO, KD or inhibition. So far JPT2 KO and Lsm12 KO showed inhibition of endolysosomal translocation and cellular infectivity but further experiments are needed to investigate the phenotypes. Comparing the respective KOs also provides an opportunity to decipher between NAADP and PI(3,5)P_2_ mediated effects on immune and other cells. Likewise, the effect of modulation of OCaR/TMEM63 proteins or Rab7a on inflammatory mediator release, phagosome resolution, phagocytosis, granzyme B secretion, or histamine release in immune cells requires more attention in the context of adenine nucleotides but also phosphoinositides.
